# Teledermatology and Inflammatory Skin Conditions during COVID-19 Era: New Perspectives and Applications

**DOI:** 10.3390/jcm11061511

**Published:** 2022-03-10

**Authors:** Claudio Marasca, Maria Carmela Annunziata, Elisa Camela, Adriana Di Guida, Luigi Fornaro, Matteo Megna, Maddalena Napolitano, Cataldo Patruno, Luca Potestio, Gabriella Fabbrocini

**Affiliations:** 1 Section of Dermatology, Department of Clinical Medicine and Surgery, University of Naples Federico II, 80126 Napoli, Italy; marica.annunziata@hotmail.it (M.C.A.); elisacamela@gmail.com (E.C.); adriana.diguida@gmail.com (A.D.G.); fornaroluigi95@gmail.com (L.F.); mat24@libero.it (M.M.); potestioluca@gmail.com (L.P.); gafabbro@unina.it (G.F.); 2 Department of Health Sciences Vincenzo Tiberio, University of Molise, 86100 Campobasso, Italy; maddy.napolitano@gmail.com; 3 Department of Health Sciences, University Magna Graecia of Catanzaro, 88100 Catanzaro, Italy; cataldo.patruno@unicz.it

**Keywords:** telemedicine, telecare, tele health, telehealth, mobile health, mHealth, m-Health, eHealth, e-Health, psoriasis, acne, hidradenitis, hidradenitis suppurativa, atopic eczema, atopic dermatitis

## Abstract

Background: The most frequent inflammatory skin diseases are psoriasis, atopic dermatitis, hidradenitis suppurativa, and acne. Their management is challenging for dermatologists since their relapsing chronic clinical course is associated with a great impact on quality of life. Nevertheless, the recent introduction of novel therapies, such as biological drugs and small molecules has been changing the history of these diseases. Methods: A systematic review of the scientific literature of case reports, case series, epidemiological studies, reviews, and systematic reviews regarding teledermatology and inflammatory skin disease. Studies were identified, screened, and extracted for relevant data following the PRISMA (preferred reporting items for systematic reviews and meta-analyses) guidelines. Results: A total of 69 cases articles were included in the review. Conclusions: As we have shown in the review, several experiences of teledermatology for patients affected by inflammatory skin diseases have been demonstrated to increase due to clinical access to hospital and specialized health care services, allowing better access to specialized dermatology care for people living in remote areas, and saving costs and money with health care.

## 1. Introduction

The most frequent inflammatory skin diseases are psoriasis, atopic dermatitis, hidradenitis suppurativa, and acne [1,2,3,4]. Their management is challenging for dermatologists since their relapsing chronic clinical course is associated with a great impact on quality of life. Nevertheless, the recent introduction of novel therapies, such as biological drugs and small molecules, has changed the history of these diseases. The COVID-19 outbreak has completely overturned our daily routine, and lockdowns established by governments made it more difficult to access health care [5]. The physicians’ answer to health issues during the pandemic was telemedicine. Although telemedicine was introduced several years ago, the worldwide spread of the SARS-CoV-2 infection at the end of 2019 allowed for the worldwide spread of teledermatology, as well. Indeed, it has gained a key role, especially in the management of patients affected by chronic inflammatory skin diseases. 

The global diffusion of telemedicine can lead to clinical improvements for both providers and patients. The aim of this review is to describe and analyze the role of telemedicine in conjunction with the most common inflammatory skin diseases.

## 2. Materials and Methods

A systematic review of the scientific literature of case reports, case series, epidemiological studies, reviews, and systematic reviews regarding teledermatology in inflammatory skin disease.

Studies were identified, screened, and extracted for relevant data following the PRISMA (preferred reporting items for systematic reviews and meta-analyses) guidelines.

**Figure 1:**
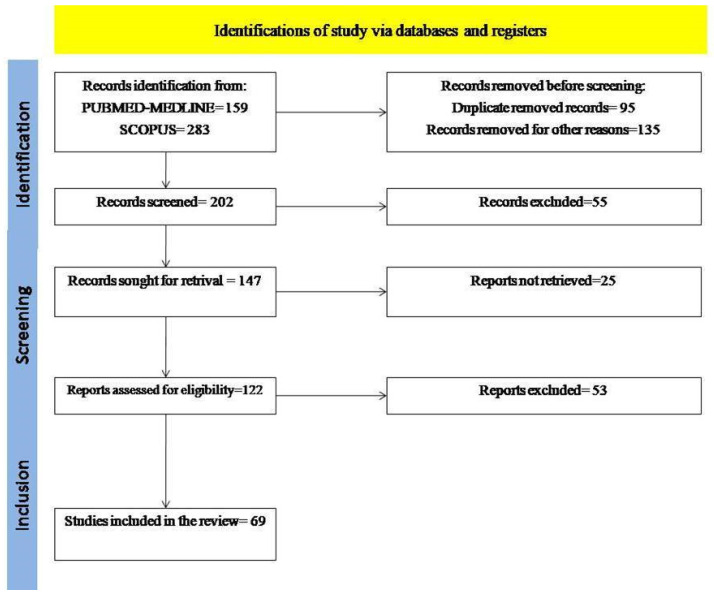
Flow chart of study analysis.

A flow chart of the systematic literature search was according to PRISMA (Figure 1).

The study was registered in PROSPERO; the registration number is 304274.

In accordance with the 2020 edition of Preferred Reporting Items for Systematic Reviews and Meta-analyses guidelines, a systematic search was performed using PubMed, Scopus, from their inception to 30 July 2021, using medical subject headings (MeSH) terms (if applicable), and medical terms for the concepts of teledermatology in inflammatory skin conditions.

In regards to the review of the literature, a search of the PubMed and Scopus databases (until July 2021) was performed, using the following research terms: “telemedicine”, “tele care”, “tele health”, “telehealth”, “mobile health”, “mHealth”, “m-Health”, “eHealth”, “e-Health”, “psoriasis”, “acne”, “hidradenitis”, “hidradenitis suppurativa”, “atopic eczema”, “atopic dermatitis”

Search criteria were the following:

(a) Articles published in scientific journals included in MEDLINE or SCOPUS databases;

(b) Articles that were written in English and all types of epidemiological studies were included: Title and abstract reviews were performed. Therapies that could be categorized as traditional Chinese medicine, herbal medicine, or Ayurveda/Ayurvedic medicine have been excluded. Articles regarding other inflammatory skin diseases were excluded. Letters, comments, reviews of telemedicine in other diseases, and papers in which, according to the opinion shared by all the authors, there was no relevant information, were not considered. The article is based on previously conducted studies. Each author independently analyzed all the articles and data. A total of 69 were selected for the evaluation in the present review.

## 3. Results

A total of 69 articles were included in the review. A summary of the included articles is shown in Table 1. For all the considered cases, data about teledermatology in specific skin conditions was available.

Papers for each skin condition were analyzed in the following paragraphs.

### 3.1. Psoriasis

Psoriasis is a chronic relapsing disease that requires long-term treatments and frequent follow-ups [6]. Besides in-person consultations, teledermatology has become an opportunity in daily clinical settings for those who, for any reason, cannot attend the visit and at the same time require medical advice [7,8,9,10,11,12,13,14,15]. In fact, Anderesen et al., through a retrospective review of the teledermatology database in the Faroe Islands, a group of 18 islands in the North Atlantic, concluded that teledermatology is indispensable in specific contexts, such as rural areas, while in non-rural ones, should be destined to evaluate which disease can benefit from either in-person or online visits to give selectivity on resources destined for telemedicine [12]. Moreover, since the worldwide spread of the SARS-CoV-2 infection at the end of 2019, telemedicine has gained an increasingly important role in daily clinical practice, especially regarding patients affected by chronic diseases, such as psoriasis [16,17]. Villani and colleagues implemented an already existing teleconsultation service that was originally reserved for emergencies, and that turned routine during the COVID-19 pandemic, given the need to avoid social contacts [13]. Such measures appeared to be very important, particularly for those patients under immunosuppressive drugs that were at high risk of contracting the infection [13]. In general, Beer, Chambers, Pearlman, and Frühauf accordingly reported in their studies that online interactions were well accepted by patients and highly preferred to in-person visits [16,17,18,19]. Moreso, Yi et al. noticed a slight decrease in access to telehealth services for elderly and non-English speaker patients, suggesting potential unequal access to care for these vulnerable patients [20]. Favoring points for teledermatology were reported to be the easy accessibility, safety, and effectiveness of teleconsultations that were perceived as patient-centered, and the possibility of saving money and time [14,20,21,22]. As a result, patients’ compliance with treatment was shown to be high, and thus, a likely unnecessary worsening of psoriasis severity, given the unattended visits for control and renewal of treatments, was prevented, as reported by Brunasso et al. [15]. Likewise, telemedicine for psoriasis was appreciated by doctors, as it reduced the burden of visits that could be managed remotely, as well as saving time and money [17,18,19,20,21,22,23]. Moreover, there has been a great push towards digitalization that involved both patients and health care systems, giving teledermatology prospects for being part of clinical practice in the future [17]. The importance of telemedicine in assessing and monitoring psoriasis and its treatment was already shown previously by Julia Frühauf et al., who ran a pilot study comparing the data of psoriasis severity and therapeutic outcomes of 10 patients under etanercept derived from face-to-face and online visits [24]. They found no statistically significant differences between the assigned scores and, accordingly, therapeutic decisions in the two settings supported the feasibility of a remote follow-up of psoriasis patients [24]. Such findings were also confirmed by Balato et al., which demonstrated the usefulness of telemedicine in improving clinical outcomes and achieving better disease control by developing an educational and motivational support service through the reliance of text messages in guiding and reminding psoriasis patients about their treatment [25]. Moreso, online consultations raised the issue of accurately determining psoriasis severity on which disease progression evaluation and therapeutic decisions are based. In fact, either the quality of cameras, pictures, video calls, or internet connection as well as patients’ ability, may have impaired the assessment of the daily conventionally adopted psoriasis severity scores, such as the Psoriasis Area and Severity Index (PASI) or body surface area (BSA). For this reason, many authors tried to address such topics in different ways. In detail, Armstrong and colleagues developed an effective, innovative, and collaborative health model where patients were directly involved in their disease monitoring through self-calculating the PASI and BSA scores after appropriate training [23]. Moreso, Singh and colleagues ran the tele-PASI accuracy study with the aim to assess the reliability of PASI scores determined on the basis of standardized digital images—the tele-PASI score [26]. Twelve patients with confirmed psoriasis were recruited, as well as two dermatologists for a baseline face-to-face visit, and a third independent dermatologist who determined the remote PASI scores at weeks 6 and 14 [27]. Both intra- and inter-observer assessments were performed, showing a good agreement in PASI score determination between the three dermatologists as well as per each one at different times, confirming analogous findings reported by Julia Furhauf et al. [24]. Furthermore, some authors wondered about the accuracy of a remote determination of conventional psoriasis severity scores, given the specific virtual evaluation. For this reason, Wu et al. developed and validated a new model to estimate a total PASI score in the context of teledermatology (the tele-PASI score) where some characteristics of psoriasis, such as thickness, cannot be assessed, in contrast to erythema, scaling and affected areas of the body [27]. Hence, the tele-PASI score was the result of the original PASI, excluding thickness [27]. An amount of 3866 patients with moderate-to-severe plaque psoriasis were included in the study and randomized into three treatments groups: placebo, ixekizumab, and etanercept [27]. A strong correlation between original and modeled total PASI scores, either at screening, baseline, or during treatment up to week 12, was found, irrespective of treatments, showing that such a proposed modified score may be safely integrated into telematic clinical practice [27]. Nonetheless, further studies are needed in support of such findings. Concerning psoriasis treatment monitoring, teledermatology has been proven to be useful in prescribing and monitoring not only systemic therapies, i.e., biologicals, but also topicals and phototherapy, thus improving patients’ adherence to treatment and clinical outcomes [28,29,30,31,32]. Moreso, telemedicine assisted psoriasis patients not only with the cutaneous aspect of the disease but also on a psychological level [33]. Indeed, Young and colleagues ran a 12-month randomized controlled equivalency trial to assess the impact of teledermatology on psoriasis patients’ mental health and depression, finding no statistically significant differences compared to in-person care [33]. Moreover, even if the adult population is the main recipient of telemedicine, studies have also shown that children were directly involved in developing awareness of their disease and evolution during the COVID-19 pandemic [34]. In general, based on doctors’ perspectives, teledermatology has been proven effective and reliable as it allows to perform visits, maintain continuative medical assistance and improve clinical outcomes; from a patients’ perspective, it helps keep contact with physicians, enhancing straightforward communications, increasing compliance to treatment and moreover, is highly preferred, as it is time and money-saving, giving the feeling of a more free, flexible and empowered lifestyle [16,18,19,21]. Hence, we believe that telemedicine will keep an important role in daily health settings, especially with a complementary role in the follow-ups of chronic diseases, such as psoriasis. Such an idea is in line with Gisondi et al., that supports the use of online consultations for patients with stable psoriasis on maintenance treatment with biological agents, as routine visits usually end up with a confirmation of ongoing treatment—different from naïve or unstable patients who would rather benefit from in-person visits [14]. Likewise, Dahy et al. would encourage face-to-face visits for establishing a diagnosis, and online consultations for the follow-ups [6]. More applications will be found with the incorporation and implementation of digital platforms in health care systems. An emerging role of teledermatology for psoriasis is the possibility for health personnel to discuss the most difficult cases through dedicated social media, improving knowledge and ultimately patients’ clinical outcomes [35].

### 3.2. Acne and Hidradenitis Suppurativa

Acne and hidradenitis suppurativa (HS) are chronic, inflammatory, and often debilitant skin conditions, strongly affecting the quality of life of patients, and requiring targeted therapies and continuous follow-up [36,37,38].

In particular, during the COVID-19 pandemic period, new therapeutic and clinical approaches have been required in order to continue the diagnostic and treatment pathways for these pathologies, minimizing the risk of infection for both the patients and the clinicians and ensuring continuous monitoring of the disease [39].

Several modalities of teledermatology services, such as video calls, phone calls, WhatsApp and Facebook support groups, image evaluation, and emails have been developed during the COVID-19 pandemic period in order to minimize the impact of COVID-19 restrictions on the quality of life and quality of treatment in patients with HS and acne.

However, even if several studies reported the use of telemedicine in patients with acne, [40,41,42,43,44,45,46,47,48,49,50,51,52] the role of remote assessment for HS patients was not extensively investigated during the COVID-19 pandemic period [49,50,51,52].

Although Brunasso et al. showed the effectiveness of email and calls in preventing HS worsening or treatment discontinuation on 11 patients with HS, the efficacy of telemedicine in patients with HS has conflicting results and many concerns [15]. In fact, Patel reported the results of a retrospective analysis conducted from April to October 2020 on 41 patients attending 73 remote consultations, compared to 40 patients attending 70 face-to-face examinations [48]. Although the efficacy of telemedicine was confirmed despite the absence of a real examination, their results suggested the importance of clinical face-to-face assessment, since HS is usually an unstable disease and photographic or video evaluation should be handled sensitively due to the high prevalence of anxiety and depression in the affected subject, and the frequent involvement of intimate body areas [49].

Moreover, a survey conducted on Facebook groups supporting HS involving 335 respondents showed that patients with a higher Hurley stage disease disagreed that telemedicine provided equally effective care for their disease, compared to in-person clinic visits (182/335, 54.32%) [50].

In conclusion, different strategies in remote care are needed in patients with HS while considering the impact on quality of life and the frequent involvement of intimate body areas [51,52].

In regards to acne disease, the role of telemedicine in acne management was also investigated before the COVID-19 pandemic period [40]. In fact, Frühauf et al. reported the results of a randomized controlled trial with the purpose of assessing the superiority of mobile teledermatology in the care of patients with severe acne compared to outpatient services. Sixty-nine patients receiving oral isotretinoin were enrolled and randomized into either the teleconsultation or the outpatient consultation arm of the study for 24 weeks. This trial showed that teledermatology was a useful, effective, safe, and well accepted tool [39]. This approach has been strongly investigated during the COVID-19 pandemic period since acne is the main condition reported to be managed through teledermatology [40]. The use of teledermatology impacted the therapies and clinical assessment of patients with acne as well as patients’ features, which affected the efficacy of this approach.

In regards to therapies, Kazi et al. reported that biologics and immunomodulators were more commonly prescribed through synchronous than asynchronous teledermatology, defined as patient-to-physician store-and-forward visits in which a patient completed a questionnaire and supplied three images of their skin lesions [41]. Kazi et al. suggested that asynchronous telemedicine should be used for acne management, while synchronous teledermatology was preferable for severe forms of this disease [41]. A recommendation in the therapeutic approach has been reported by Gu et al. as well [42]. Indeed, even if the effectiveness of acne management through telemedicine was confirmed in their study, reporting their experience on 480 video visits, the authors advised clinicians to be careful in the use of isotretinoin in female patients if visits are conducted in telemedicine since there are no studies assessing the efficacy and safety of teledermatology in this population [42]. Moreso, treatment adherence could be improved through telemedicine. In fact, Marasca et al. showed the importance of the WhatsApp supporting group in acne patients, reporting that the 80 subjects who received messages reminding them of acne medications had increased therapeutic adherence and better outcomes compared to the group of 80 patients not receiving medical advice in the 12-week study [43].

Regarding patient satisfaction and outcome with telemedicine, Ruggiero et al. showed the satisfaction of patients after a teledermatological visit in an observational prospective study, reporting patient experience and feeling with this technique, [44] as well as Villani et al., which reported the effectiveness of video consultants to assess acne severity, reducing treatment delay and in-person visits [45].

However, the need for a targeted teledermatological approach has been reported by Lee et al., who demonstrated that non-English speaking and elderly patients were more likely to use audio-only than video visits compared with the English speaking and younger patients, examining 1233 virtual visits conducted from March to May 2020. Their results suggested the importance of non-video telemedicine alternatives for these populations [46].

Finally, a comparison between patients seen via teledermatology and patients seen via face-to-face evaluation showed that the median time to follow-up was 45.5 days in the teledermatology group compared to 64 days in the face-to-face group (*p* < 0.001) and that oral antibiotics (43.0% versus 28.5%) were more used in teledermatology patients compared to face-to-face patients (*p* < 0.001), showing the need to improve teledermatology follow-up education and follow-up care [47].

All these reported studies confirmed the effective adherence to treatment and health-related quality of life of telemedicine in current clinical practice, also considering and overcoming the difficulties that this approach may have. Moreover, other studies are ongoing to refine this technique, and new scores are under investigation to evaluate acne severity through the use of images and telemedicine [52].

### 3.3. Atopic Dermatitis

Atopic dermatitis (AD), also known as atopic eczema, is a chronic inflammatory skin condition characterized by intense itch, disruption of the skin barrier, and upregulation of type 2-mediated immune responses in the skin [53]. AD is considered one of the most common chronic conditions, affecting 15% to 30% of children and 2% to 10% of adults, with a global prevalence of nearly 230 million [54]. Over the past 30 years, there has been a significant burden of AD cases worldwide, with a rapid increase, especially in developing countries [55]. Furthermore, the early onset in childhood, the chronic course, the need for specialist visits, and the frequent atopic comorbidities make AD an important global public health issue [4]. In this context, telemedicine has set out to provide diagnostic and management solutions for AD, thereby optimizing the supply of in-person appointments with dermatologists for a lot of severe cases [56]. The European Academy of Allergy and Clinical Immunology concerning AD proposed that telemedicine could be useful for the monitoring of the severity of disease (using validated instruments for scoring the severity of AD in a mobile app), therapeutic education, patient communication, medication reminders, and research [57]. In the last years, the importance of telemedicine as a valid educational approach in supporting AD patients has been evaluated in some clinical studies [58,59,60,61].

Bergmo et al. have conducted a randomized controlled trial to analyze how web-based consultations for parents of children with AD affected self-management behavior, health outcome, health resource use, and family costs [58]. Patients were randomly assigned to intervention and control groups. The intervention group received remote dermatology consultations through a secure web-based communication system [58]. The control group was encouraged to seek treatment through traditional means, such as general practitioner visits and hospital care [58]. Both groups received a 30-min individual face-to-face educational session prior to the intervention [58]. After one year, no change in self-management behavior, health outcome, or costs has been found [58]. The intervention group tended to have fewer visits to practitioners offering complementary therapies than the control group, with a positive correlation between emergency visits at baseline and messages sent. Both groups, however, reduced the mean number of skincare treatments performed per week and had fewer total health care visits after the intervention [58].

The effects of online courses in continuing medical education were evaluated by Schopf et al., showing that this strategy could be a valid tool for the training of doctors and nurses, with the advantage of reducing costs related to travel expenses [59]. Recently, Giavina-Bianchi et al. have reported results of a retrospective study in a population of 30,976 individuals assisted by telemedicine [60]. Their aim was to evaluate the proportion of AD subjects who could be managed with the support of telemedicine and its accuracy [60]. AD was diagnosed in 5.3% of patients with an accuracy of 84.4%. Primary care physicians were able to manage 72% of the atopic patients, whereas 28% of them were referred to dermatologists [60]. Additionally, telemedicine is effective as usual face-to-face care about the quality of life and severity of disease, but with the advantage of a substantial cost-saving [61,62,63,64].

Furthermore, a universally recognized power of telemedicine is the ability to reduce distances and reach places where health resources are scarce [65,66]. Indeed, the position statement on AD in sub-Saharan Africa argues that the potential applications of telemedicine include its use for atopic schools, e-learning, webinars with AD experts with local knowledge of local drugs and practices, pigment-adapted apps, skin tutorial videos, and therapeutic education for patients and families [65]. Certainly, the COVID-19 pandemic has given a great implementation to the spread and use of telemedicine, allowing efficient diagnostic-therapeutic management in patients suffering from AD [15,67,68,69]. Telehealth consultations were useful for triage of the severity of the disease, ensuring in-person visits only for the severe cases, and remote monitoring of mild and moderate cases [68]. Indeed, during the COVID-19 pandemic, several approaches have been adopted, such as phone calls, e-mails, e-drug prescriptions, and e-lab prescriptions [15]. Napolitano et al., for example, used telephone consultations with adult AD patients treated with dupilumab to prevent patients from leaving their homes and crowding the hospital and to continue monitoring their condition [69].

An important aspect of telemedicine is the protection of privacy and, for this purpose, standardized digital platforms should be provided to patients and health care professionals [70]. When these methods are not accessible, more common tools could be used, providing adequately informed consent acquired from the patient for the treatment via telemedicine [70].

**Table 1:** Summary of papers.

Authors	Year	Country	Objectives	Main Findings
Dahy et al. [6]	2020	Egypt	To collect the evidence regarding the efficacy of telemedicine in psoriasis management.	Telemedicine (alone or in combination) had the same or higher efficacy of psoriasis management compared to usual care.
Muir et al. [7]	2014	Australia	To explore the use and potential of store-and-forward teledermatology in Australia.	The store-and-forward teledermatology increases availability and reduces time, cost, and professional isolation. Anyway, uptake is low, for lack of awareness, increased workload for referring practitioners, and lack of financial incentives.
Doraiswamy et al. [8]	2020	Qatar	To collect literature on telehealth during the COVID-19 pandemic.	Increasing literature concerning telehealth, especially from high-income countries, was reported during the first 6 months of the COVID-19 pandemic. Anyway, it should be prompted in low/middle income countries and resource-limited settings.
Byrom et al. [9]	2016	Australia	To identify the current scope of Tele-Derm, the types of dermatological complaints experienced in the rural primary care setting, and to assess the quality of patient clinical information provided to the dermatologist.	The most common dermatological complaint in rural settings was dermatitis. Children represented 1/3 of patients. The average time from submission to dermatologist’s reply was 5.5 h. Clinical photos were provided in most of the cases and displayed good quality. Management advice was provided in most cases, of which refers to the case-based learning modules on Tele-Derm was made in 21% of cases. Patient outcome was largely unknown (83%). Tele-Derm can be used as an adjunct to advice provided to rural doctors seeking advice for patient management.
Jemec et al. [10]	2008	Denmark	To show the use of telemedicine as an adjunct to conventional clinical dermatology on the Faroe Islands.	The service is based on a nurse-led dermatological clinic, and teledermatology is combined with specialist visits for more complex diagnoses or procedures.
Tsang et al. [11]	2011	USA	To characterize the conditions diagnosed through clinicopathological correlation in conjunction with photos and tissue submitted to the African Teledermatology Project.	Clinical images may not be sufficient to make a diagnosis through telemedicine consultation. Histological report of skin biopsies is an important aid for diagnosis of disease and their treatment.
Andersen et al. [12]	2019	Denmark	To review the teledermatology database of the Faroe Islands from its inauguration in 2003 to November 2018.	Teledermatology is a useful tool for dermatological conditions that do not require in-person visits in non-rural contexts; anyway, it is the only available alternative in rural areas for any kind of dermatological disease.
Villani et al. [13]	2020	Italy	To show the measures applied in the dermatologic clinic of the University of Naples Federico II, Italy, to ensure a continuous follow-up, especially for chronic skin diseases (psoriasis, acne, and hidradenitis suppurativa) during the COVID-19 pandemic.	The following services were implemented: phone consultations, video consultations through teledermatology service, and WhatsApp support group.
Gisondi et al. [14]	2021	Italy	To investigate the preference for telemedicine versus in-person visits among patients with psoriasis under biological drugs and the reported reasons behind their preferences.	About 50% of patients preferred telemedicine to an in-person visit, and the main reported reasons were saving time and safety in relation to the risk of SARS-CoV-2 infection. By contrast, the main reason to prefer in-person visits was the inability to use video-communication devices.
Brunasso et al. [15]	2020	Italy	To report the experience with teledermatologic services in smartworking using phone calls and emails for chronic skin diseases at the Department of Dermatology, Galliera Hospital, Genoa, Italy.	This real-life experience showed that remote monitoring was effective in preventing unnecessary worsening of severe chronic skin diseases and poor outcomes due to the withdrawal of current therapy.
Beer et al. [16]	2021	USA	To identify dermatological diseases that may be suitable for teledermatology versus in-person visits.	Psoriasis acne and atopic dermatitis may be managed through teledermatology. Anyway, also skin cancers may be included in the future, provided the availability of new technologies to allow the diagnosis, surveillance, and treatment.
Chamber et al. [17]	2012	USA	To compare the clinical equivalence of a novel patient-centered online health care delivery model with standard in-office care for follow-up treatment of patients with psoriasis.	PASI, DLQI, IGA improved in both online and in-office groups without significant differences. Hence, a patient-centered online model may be an effective alternative to in-office care for follow-up management of psoriasis.
Pearlman et al. [18]	2021	USA	To evaluate patients’ attitudes toward synchronous teledermatology.	Included patients were familiar with social media platforms (Facebook, hardware platforms, and apple device). About 90% of patients were satisfied with synchronous teledermatology and did not find any technical difficulty during the consult. Anyway, the majority preferred in-person visits. Thus, synchronous teledermatology allows patients to access specialty consultation. It is well-received and appreciated by patients despite technical barriers, especially during a global health crisis.
Frühauf et al. [19]	2012	Austria	To evaluate the acceptance of mobile teledermatology for the home monitoring of high-need patients with psoriasis.	Mobile teledermatology is a precious tool for the home monitoring of patients with psoriasis. Moreover, it is well accepted by both patients and physicians.
Yi et al. [20]	2021	USA	To evaluate a new practice model represented by a hybrid model of in-person and teledermatology visits at a 90% to 10% ratio, to identify trends in access to and quality of teledermatology services.	With in-person visits halted for the COVID-19 pandemic, there was a decrease in elderly and non-English-speaking patients seeking teleconsultations in dermatology, so they may experience unequal access to care.
Ferwerda et al. [21]	2013	Netherlands	To evaluate perspectives of patients with psoriasis and rheumatoid arthritis towards internet-based cognitive-behavioral treatments (CBT).	Patients appreciated the CBT as time-saving and easy to use. Anyway, the inability to use a computer and the lack of face-to-face interaction with the therapist may be a disadvantage. Hence, from the patients’ perspective, internet-based CBT is a promising health care development.
Adam et al. [22]	2019	USA	To evaluate the impact of an online, collaborative connected-health (CCH) model on psoriasis management on access to specialty care.	The CCH model resulted in less distance traveled and transportation as well as in-office waiting time compared to in-person care. Both patients and providers were highly satisfied with CCH. The CCH model resulted in increased access to specialty care and enabled patient-centered, safe, and effective management of psoriasis patients.
Armstrong et al. [23]	2018	USA	To determine if an online, collaborative connected-health model results in equivalent clinical improvements in psoriasis compared with in-person care.	The online, collaborative connected-health model was as effective as in-person management in improving clinical outcomes among patients with psoriasis.
Frühauf et al. [24]	2015	Austria	To evaluate the feasibility of teledermatology services for high-need patients with psoriasis.	The patient-driven mobile home monitoring system was feasible for high-need patients with psoriasis and actively involved them in their treatment process. Moreover, there was a strong correlation between psoriasis severity measurements and therapeutic management obtained during online and in-person visits.
Balato et al. [25]	2013	Italy	To evaluate the use of text message (TM) in improving treatment adherence and patient outcomes, such as quality of life, disease severity, patient-perceived disease severity, and the patient-physician relationship.	TM increases patients’ adherence to treatment and improves self-care and patient-physician relationships, allowing improved clinical outcomes and better control of the disease.
Singh et al. [26]	2011	Australia	To assess the feasibility of the remote determination of PASI scores by comparing the results of face-to-face with digital image assessment.	PASI scores can be determined with moderate-to-good accuracy by dermatologists using standardized digital images. Thus, the implementation of a tele-PASI service may act as an adjunct to the care of patients with severe psoriasis that are unavailable for face-to-face consultations.
Wu et al. [27]	2021	Australia	To develop and validate a model for estimating total PASI score for assessment in teledermatology (Tele-PASI).	A strong correlation between the tele-PASI scores and the original total PASI scores at baseline and during treatment was reported. Hence, a tele-PASI score may be useful in determining psoriasis severity in teledermatology settings.
Koller et al. [28]	2011	Austria	To investigate the applicability of a mobile phone-based teledermatological system for monitoring psoriasis patients on biologic therapy.	The mobile phone-based teledermatological system is useful for the long-term monitoring of patients with psoriasis under systemic therapies, such as biological drugs.
Klotz et al. [29]	2005	Canada	To investigate the use of telemedicine in the monitoring of phototherapy of psoriasis patients living in a Nova Scotia region with no dermatologist.	Telemedicine provided an excellent way to monitor patients receiving phototherapy in a region without dermatologists. Overall, patient care improved. More patients were treated effectively, with better outcomes and fewer side effects.
Svendsen et al. [30]	2018	Denmark	To review randomized controlled trials (RCTs) testing eHealth interventions designed to improve adherence to topical antipsoriatic agents and to review applications for smartphones (apps) incorporating the word psoriasis.	An improvement in medical adherence and reduction in the severity of psoriasis was reported. A total of 184 apps contained the word psoriasis.
Rompoti et al. [31]	2019	Greece	Primary endpoint was the percentage of patients who achieved a PASIreduction of 75% (PASI75) at Week 16.	An improvement in the PASI index.
Li et al. [32]	2020		To assess through phone calls the consequences of infliximab interruption in psoriatic patients during the COVID-19 pandemic.	The majority of patients had lesions exacerbation and anxiety, so it is recommended to administer common drugs for psoriasis at home. Telemedicine should be advocated as a main medical visit mode during the outbreak of COVID-19.
Young et al. [33]	2019	USA	To evaluate how a novel online health model that facilitates physician-patient collaboration compares with in-person care for improving psoriasis patients’ functional status and mental health.	The online health model was equivalent to in-person care for reducing functional impairment and depressive symptoms in psoriasis patients.
Beytout et al. [34]	2021	France	To investigate the impact of the COVID-19 pandemic on children with psoriasis.	This study demonstrates the impact of the COVID-19 pandemic on children with psoriasis. Overall, psoriasis worsening, visit cancellation and fear to attend visits were reported. Moreover, patients felt that their psoriasis did not allow them to respect the hygiene measures, such as using an alcoholic solution, wearing the mask, and hand washing. Teleconsultations played a key role in patient management as regards patient monitoring, provision of information, and renewal of treatments.
Mazzuoccolo et al. [35]	2019	Argentina	To evaluate the implementation of a WhatsApp discussion group in Project ECHO (Extension for Community Health care Outcomes) Psoriasis in Argentina.	The Project ECHO Psoriasis was shown to be promising in reducing the gap of knowledge, promoting better clinical decisions through the empowerment of physicians working in remote areas.
Frühauf et al. [39]	2015	Austria	To examine the superiority in terms of effectiveness, safety, and patient compliance of mobile teledermatology in the care of patients with high-need facial acne in comparison to outpatient services.	Teledermatology is a useful tool among patients with high-need acne. It could be a valuable adjunct to outpatient care services.
Loh et al. [40]	2021	Singapore	To evaluate the implementation of teledermatology.	Teledermatology is an excellent tool for dermatoses management during the COVID-19 pandemic.
Kazi et al. [41]	2021	USA	To show quantitative data about the use of teledermatology.	The use of asynchronous teledermatology was preferred for acne management, whereas synchronous teledermatology was preferable to providers for complex medical dermatology.
Gu et al. [42]	2021	USA	To evaluate the features of acne visits during the pandemic.	Even if face-to-face visits were permitted, the majority of systemic acne management and about half of acne visits were conducted in telemedicine.
Marasca et al. [43]	2020	Italy	Evaluate the effectiveness of short message service to follow-up some patients.	The SMS group, who received the daily medical support by text, showed an increased treatment adherence.
Ruggiero et al. [44]	2020	Italy	To evaluate patients’ experience of teledermatology visits	Most of the patients (92.3%) appreciated the visits and the treatment received.
Villani et al. [45]	2020	Italy	To show the experience about the use of teledermatology for patients affected by acne disease.	Even if telemedicine is related to video or images quality, and to patients’ compliance, it could be an important supportive tool.
Lee, M. al. [46]	2020		To show the experience about the use of teledermatology for patients affected by acne disease	Even if teledermatology is a promising tool to extend dermatologic care with earlier access to follow-up., it could be useful in acne patients
Khosravi et al. [47]	2020	USA	To compare the rate and duration of follow-up between acne patients initially evaluated by teledermatology to in-person outpatient consultation.	Even if teledermatology is a promising tool to extend dermatologic care with earlier access to follow-up., follow-up education needs to be improved to improve follow-up care.
Patel et al. [48]	2021	UK	To evaluate the effectiveness of teledermatology on the care of patients with HS during the pandemic.	Face-to-face consultations should be preferred, as HS patients should be handled sensitively, given the propensity of the disease to affect intimate body areas and the possibility of anxiety and depression in these patients.
Price et al. [49]	2021	USA	To evaluate the impact of COVID-19 on HS care.	Teledermatology is a useful tool for HS management. However, an improvement of this strategy is needed to optimize care for HS patients.
Kang et al. [50]	2020	Canada	To examine teledermatology management strategies and treatment outcomes.	Patients affected by HS need special strategies in telemedicine. Support group should be considered. More data is needed to improve HS management in telemedicine.
Foolad et al. [52]	2017	USA	To assess the difference in acne grading and treatment recommendations among an international group of dermatologists evaluating photographs.	The study suggests the effectiveness of the use of mobile phone-based photography and cloud-based image sharing for acne assessment.
Ariens et al. [56]	2017	Netherlands	To assess opinions of the most important stakeholders influencing the implementation and use of eHealth services in daily dermatology practice	Health care professionals and patients acknowledge the benefits arising from the implementation and use of eHealth services in daily dermatology practice.
Matricardi et al. [57]	2019	EAACI Task force	To evaluate the current and future potential of mHealth for specific areas of allergology, including allergic rhinitis, aerobiology, allergen immunotherapy, asthma, dermatological diseases, food allergies, anaphylaxis, insect venom, and drug allergy.	The perspectives of health care professionals and allergic patients are discussed, underlining the need for thorough investigation for an effective design of mHealth technologies as auxiliary tools to improve the quality of care.
Bergmo et al. [58]	2009	Norway	To analyze how web-based consultations for parents of children with atopic dermatitis affect self-management behaviour, health outcome, health resource use, and family costs.	No effect of supplementing traditional treatment for childhood dermatitis with web-based consultations was found, however, the intervention group tended to have fewer visits to practitioners.
Schopf et al. [59]	2012	Norway	To investigate whether an interactive Web-based course, including personal guidance via email or cellular phone texting, may be used to improve practice behavior of general practitioners in the management of atopic dermatitis.	A Web-based educational intervention aimed at general practitioners combined with personal support can reduce the number of atopic dermatitis patient referrals to specialists.
Giavina-Bianchi et al. [60]	2020	Brazil	To evaluate the proportion of atopic dermatitis patients who could be managed with the support of telemedicine and its accuracy. To assess the frequency of atopic dermatitis, demographics, clinical features, and therapies dispensed in relation to the disease.	Telemedicine was an accurate method and helped primary care physicians to treat 72% of the atopic dermatitis lesions, optimizing in-person appointments with dermatologists for more severe cases.
van Os-Medendorp et al. [61]	2012	Netherlands	To determine the cost-effectiveness of individualized e-health compared with usual face-to-face care for children and adults with AD.	E-health during follow-up of patients with AD is just as effective as usual face-to-face care with regard to the quality of life and severity of disease, with the advantage of cost-saving.
Kornmehl et al. [62]	2017	USA	To evaluate the quality of life in AD patients managed through a direct-access online model.	Adult and pediatric AD patients receiving direct-access online care had an equivalent quality of life outcomes as those seen in-person. The direct-access online model has the potential to increase access to care for patients with chronic skin diseases.
Santer et al. [63]	2014	United Kingdom	To develop and test a Web-based intervention to support families of children with eczema, and to explore whether support from a health care professional (HCP) is necessary to engage participants with the intervention	This trial demonstrates the potential for greater improvements in POEM scores in website intervention groups. Moreover, HCP support was not strongly valued by participants and did not lead to better outcomes.
Armstrong et al. [64]	2015	USA	To compare the effectiveness of a direct-access online model for follow-up dermatologic care in pediatric and adult patients with atopic dermatitis with that of in-person office visits.	The direct-access online model results in equivalent improvements in atopic dermatitis clinical outcomes as in-person care.
Schmid-Grendelmeier et al. [65]	2019	Africa	To identify research and intervention priorities in Africa and initiate African-led projects for AD.	AD is one of the most prevalent chronic inflammatory skin diseases in SSA. The potential applications of telemedicine could improve the diagnosis and therapeutic management of AD.
Davis et al. [66]	2021	USA	To describe current evidence regarding health disparities within allergy/immunology in racial and ethnic underserved populations.	Health disparities affect all aspects of health care, especially with regard to access, delivery, and outcomes. Telemedicine could improve access to health care in AD patients,
Shaker et al. [67]	2020	USA and Canada	To provide useful strategies for the correct diagnostic and therapeutic management of patients referring to allergy/immunological clinics during the COVID-19 pandemic.	Telehealth and virtual patient encounters can be central to delivering allergy services within a risk-stratified context of the SARS-CoV-2 pandemic, thanks to the ability to integrate telecommunications, information systems, and patient care.
Napolitano et al. [68]	2020	Italy	To provide monitoring strategies for patients with atopic dermatitis treated with dupilumab, during the COVID-19 pandemic.	Telephone consultations with adult AD patients treated with dupilumab prevent patients from leaving their homes and crowding the hospital and to continue monitoring their condition.
Malipiero et al. [69]	2020		To evaluate the reorganization of work in allergy clinics to fight the COVID-19 pandemic	Telemedicine and digital medicine services can be helpful to reduce the risk of viral spreading while delivering up-to-date personalized care

## 4. Discussion

Our review starts with the World Health Organization (WHO) definition of telemedicine as the “delivery of health care services, where distance is a critical factor, by all health care professionals using information and communication technologies for the exchange of valid information for diagnosis, treatment, and prevention of disease and injuries, research, and evaluation, and for the continuing education of health care providers, all in the interests of advancing the health of individuals and their communities” [70]. In the WHO definition are enclosed the main objectives of telemedicine, such as providing clinical assistance remotely, evading spatial distance, and using new technologies at the service of patients without reducing the quality of health care. 

Nonetheless, to date, the definition of telemedicine has broadened, since distance is not the only reasonable motive to rely on this service anymore. In fact, many other factors may be involved, either related to patients, health care systems, or global situations.

Certainly, teledermatology fulfills an invaluable role in daily clinical settings for those who, for any reason, cannot attend the visit and at the same time, require medical advice. In fact, it allows for overcoming logistical barriers, such as geographical isolation, time constraint, and few availabilities of dermatologists in regional areas [7,8,9]. This appears to be invaluable, especially for patients living in rural, remote, or underserved areas, such as in Australia, North Africa, and islands [10,11,12,13,14,15]. Indeed, inhabitants from such areas often do not have alternatives to online consultations, and in general, health care services tend to push teledermatology as time and cost-saving [15].

Moreover, the COVID-19 pandemic has pushed the use of teledermatology as an inestimable health care service, especially for those patients affected by chronic skin diseases, such as psoriasis, acne, hidradenitis suppurativa, and atopic dermatitis, requiring continuity of care. Indeed, the need to guarantee therapeutic assistance on the one hand and preventing the infection from spreading, on the other hand, has prompted the development of telecommunications to facilitate the interactions between patients and doctors, especially during the lockdown [8,9,10,11,12,13,14]. For this reason, dermatological departments developed and boosted online services, such as email, phone, or video calls, as well as digital platform support groups to provide assistance to patients during the COVID-19 pandemic.

Nonetheless, years ago, some hospitals all over the world were already trying to develop a telehealth system, anticipating in some ways, a striking necessity during the recent lockdown. In detail, teleconsultation platforms, as well as strategies through text messages, were proposed to increase patients’ adherence to treatment [24,25] and to make access to care easier and shorter [24,25]. Furthermore, the COVID-19 pandemic accelerated the process of digitalization, both for health structures, doctors, and patients that needed to update on the e-services. Thus, in order to ensure health care continuity, video calls, phone calls, text messages, or email services were implemented all over the world in hospitals, and thus, patients could have access to care.

As shown in the present review, several teledermatology experiences for patients affected by inflammatory skin diseases have been demonstrated as increasing clinical access to hospitals and specialized health care services, allowing better access to specialized dermatology care for people living in remote areas, while saving costs and money by health care [8].

Positive aspects of teleconsultations were easy accessibility, safety, and effectiveness, as reported by patients and physicians [16,18,19,21,47]. By contrast, limiting factors to telehealth service access were reported to be the inability to use or the unavailability of internet-based platforms, as well as technological devices [16,18,19,21,47].

In the near future, teledermatology is expected to become an integral part of daily clinical practice and possibly replace in-person visits for some categories of patients or specific types of consultations. Indeed, follow-up visits of patients affected by chronic diseases in stable phases may represent the most suitable beneficiaries of this health care modality [14]. Potentially, other dermatological diseases, such as skin cancer, may also be involved, provided the development and availability of technological tools with high resolutions that would allow a reliable assessment at distance by expert dermatologists [16]. This service could be useful for other health care professionals, such as general practitioners or dermatologists that are not experts in the oncodermatological field [16].

Certainly, access to technologies, as well as the internet, are limiting and mandatory factors for teledermatology, together with the ability and the will to use them. Moreover, it is fundamental to regulate laws on patients’ privacy in the use of telehealth platforms, either synchronous or asynchronous, to protect sensitive data [16]. Finally, new parameters and scores evaluating the severity and clinical outcomes of dermatological diseases using video consultations and images are required [47].

Concluding, teledermatology is a current reality in daily clinical practice, especially in the management and follow-up of patients affected by chronic skin diseases. It is a useful tool that allows cost and time-savings both for patients and health structures. In the future perspective, teledermatology will hold a leading position in many other aspects of dermatology and types of patients.

## 5. Strengths and Limitations

The PRISMA guidelines used to review current literature about teledermatology use are the main strength of our work. Limitations include the limited data on the use of teledermatology post the COVID-19 pandemic period and the lack of studies with a large cohort to specifically analyze the effectiveness of teledermatology in psoriasis, acne, HS, and AD management.

## 6. Conclusions

Teledermatology has shown to be an effective and safe tool for the continuous monitoring and assessment of the severity and treatment of chronic inflammatory skin conditions, particularly during the COVID-19 pandemic period. Certainly, further studies are needed to improve this useful weapon for future applications.

## Data Availability

Data sharing is not applicable to this article as no datasets were generated or analyzed during the current study.
